# Promoting Whole Health in the Dental Setting: Steps Toward an Integrated Interprofessional Clinical Learning Environment Involving Pharmacy, Social Work, and Nursing

**DOI:** 10.5334/ijic.5814

**Published:** 2021-11-18

**Authors:** Kimberly A. Sanders, Lisa de Saxe Zerden, Meg Zomorodi, Katharine Ciarrocca, Karen L. Schmitz

**Affiliations:** 1 University of North Carolina at Chapel Hill, Eshelman School of Pharmacy, US; 2 University of North Carolina at Chapel Hill, Adams School of Dentistry, US; 3 University of North Carolina at Chapel Hill, School of Social Work, US; 4 University of North Carolina at Chapel Hill, Office of the Provost, US; 5 University of North Carolina at Chapel Hill, School of Nursing, US; 6 Duke University Hospital, Division of Plastic Maxillofacial, and Oral Surgery, US

**Keywords:** oral health integration, interprofessional care, behavioral health, integrated care, interprofessional education, interprofessional practice

## Abstract

**Introduction:**

Dental settings have not traditionally functioned as access points to the health care system, however they can serve patients who may not otherwise seek routine health care. Millions of Americans annually visit either a dental or primary care provider, but not always both as recommended, even though multiple health co-morbidities can manifest in and impact oral health. Offering multidisciplinary health services in a dental setting has potential to reach unserved populations.

**Description:**

Innovative partnerships between schools of dentistry, pharmacy, social work, and nursing were designed to promote integrated service delivery in the emerging workforce and the purposeful inclusion of oral health in integrated care settings.

**Discussion:**

Oral complications of systemic disease and systemic complications of oral disease impose significant burdens on populations and the public health infrastructure in terms of economic cost, disability, and mortality. Exacerbated by the lack of integrated services, intersecting social, economic, and health issues perpetuate disparities and negative health outcomes. Care is often focused on reactive rather than preventive measures therefore addressing only the acute issue instead of the underlying, causative problem(s).

**Conclusion:**

We describe steps for integrated, whole-health services and lessons learned for other academic health institutions and interprofessional settings considering integrated clinical models.

## Introduction

In the United States, a web of unmet physical, oral, behavioral health, and social needs are exacerbating the disparities in access to health services and related outcomes. Nearly 20% of the adult population do not have a routine source of health care or a medical home, including access to a primary care provider. This statistic is substantially worse among racial and ethnic minorities [1]. Although national efforts have sought to transform health systems by increasing collaboration among and integration of physical and behavioral health services, oral health has often been overlooked by these efforts [2,3]. Access to adequate and routine dental care is also a widespread national issue: as of 2017, more than 49 million Americans lived in dental health professional shortage areas [4,5]. Notably, approximately 75% of dental health professional shortage areas – defined as a population-to-provider ratio of 5,000 to 1 – are located in rural areas [6]. Given these troubling shortages and the strong correlations between oral health and chronic illness [3,7,8], there is a clear and urgent need to incorporate oral health services into new, collaborative health systems.

This paper describes how an integrated health care team was established within a school of dentistry in the southeastern United States, providing an interprofessional clinical learning environment for student learners. This innovative partnership between the schools of dentistry, pharmacy, social work, and nursing was designed to promote integrated service delivery in the U.S.’s emerging health care workforce, and the purposeful inclusion of oral health in integrated care settings. To our knowledge, no prior interprofessional learning program has integrated physical health, behavioral health, and oral health in a primarily dental care setting.

A seminal report, *Oral Health in America: A Report of the Surgeon General*, described national data trends linking oral health conditions such as periodontal (gum) disease to chronic diseases such as heart disease, diabetes, stroke, and chronic obstructive pulmonary disease [7]. These chronic diseases are leading causes of hospital readmission, but also of oral health-related complications [9,10,11,12]. There are other well-documented overlaps between oral and physical health. For instance, dental caries (i.e., cavities), the most common chronic childhood disease, disproportionately impacts children who experience asthma [13]. Children with poor oral health were nearly three times more likely than their counterparts to miss school as a result of dental pain [14]. Additionally, poor oral health among pregnant women has been associated with worse birth outcomes, specifically low birth weight babies [15]. Oral complications of systemic disease and similarly, the systemic complications of oral disease impose significant burdens on populations and the public health infrastructure in terms of economic cost [16], disability, and mortality [1,17]. Exacerbated by the lack of integrated services, these intersecting social, economic, and health issues perpetuate disparities and negative health outcomes. As a result, care is often focused on reactive rather than preventive measures by addressing only the acute issue instead of the underlying, causative problem(s) [18].

Abundant evidence demonstrates how mental illness and substance use disorders contribute to poor health outcomes and unmet treatment needs both in oral health and overall health [2,19]. Notably, this relationship is bi-directional. Many psychotropic medications cause xerostomia (i.e., dry mouth), a known risk factor for oral health diseases [20]. A 2015 systematic review comparing the oral health of people with severe mental illness (SMI) to the oral health of the general population found that people with SMI were almost three times as likely to have lost all their teeth and had significantly higher rates of decayed teeth, missing teeth, and fillings [21]. Conversely, the U.S. Surgeon General has described how oral disease contributes to negative self-image, self-esteem, and normal interactions with others, which can in turn lead to chronic stress and depression [9,22]. Furthermore, poor oral health can impact how an individual eats, their food choice, and their speech abilities – all which can affect quality of life [23]. Given the extensive interconnection of oral, physical, and mental health issues, expanding integrated care services to include oral health is a logical step toward better supporting a patient’s whole health [24].

Despite the World Health Organization’s recognition that oral health is key to overall health and an important quality of life indicator [25], oral health services are still rarely included as part of integrated service delivery. Although dental settings have not traditionally served as access points for whole-person care, multiple co-morbidities can manifest and/or impact oral health. Thus, taking advantage of the dental setting as an access point is crucial. Based on data from the American Dental Association Health Policy Resources Center, Vujicic and colleagues note that “in any given year, 27 million Americans visit a dentist but do not see a physician. Another 108 million visit a physician but do not see a dentist, including more than 60% of children aged 1 through 4 years” [19]. While separate access to medical care and dental care has not been optimized, it clearly makes the case that offering oral, physical, and behavioral health services in the same settings has great potential to reach large, unserved populations.

As health systems and educational institutions continue to make strides toward the quadruple aim of health care – improving patient outcomes, increasing efficiency, lowering costs, and preventing provider burnout [25] – coordinated and innovative responses are imperative to addressing health needs [3,8,24,27,28]. However, holistically addressing patient and population health requires seismic shifts in how care is provided and how future healthcare professionals receive integrated health care training. To this end, this paper describes: (a) the impetus for a novel, integrated, interprofessional clinical learning environment within a school of dentistry in the southeastern United States, (b) the steps taken to implement an innovative collaborative partnership between one university’s schools of dentistry, pharmacy, social work, and nursing, and (c) the strategies taken to address organizational barriers to implementation. In order to promote integrated service delivery and the purposeful inclusion of oral health services in integrated care settings, this paper offers readers a roadmap for how integrated, whole-health services can be created and details lessons learned for other academic health institutions and interprofessional clinical settings considering developing an integrated model.

## Description of the Care Practice

### The North Carolina Context—Why Integrated Care Inclusive of Dentistry is Needed

In North Carolina (NC), 74 out of the state’s 100 counties are dental health professional shortage areas; among those counties, 59 are also primary care and behavioral health shortage areas [29]. Many populations in NC also face challenges to medication management for chronic conditions due to issues related to access, affordability, lack of education, and follow-up. These shortage areas and challenges broadly jeopardize NC’s population health, as evidenced by the state’s poor health rankings in several key health indicators, including high rates of poverty [30], food insecurity, and uninsured or underinsured populations [31]. Shortage areas also impede patients’ access to services and management of myriad chronic health and social conditions. These statewide statistics reflect the daily reality of the patient population seen at the University of North Carolina (UNC) Adams School of Dentistry (ASOD).

A 2018 preliminary internal needs assessment found that of the over 100,000 North Carolinians seen in a five-year period in the Adams School of Dentistry’s student clinics, nearly 22% reported that they had not seen a primary care provider in the past two years. Furthermore, the majority of the ASOD’s patients (a) live in rural areas that are statistically likely to have provider shortages and (b) present with multiple chronic conditions, incomplete medical and medication histories, non-adherence or inappropriate medications, emotional distress, and inadequate food, transportation, and/or living arrangements. As a result, these patients often require services beyond an oral health provider’s professional scope of practice. Faced with the extensive service needs of these patients, faculty providers and student learners in the ASOD can feel inadequately prepared to meet those needs without the assistance and expertise of multiple interdisciplinary providers.

Although there are current examples of innovative programs that have integrated medical and dental care [32], pharmacy and dental care [33,34,35,36,37], or social work and oral health [38], few of these integrated models involve multiple professions collaboratively addressing the physical, oral, behavioral health, and psychosocial supports necessary for patient care in academic dental settings. To more comprehensively address patients’ needs at the ASOD at University of North Carolina and find ways to train health professions’ student learners, the ASOD needed to create new opportunities for interprofessional, integrated collaborations.

### Interprofessional Transformation

**Figure 1:**
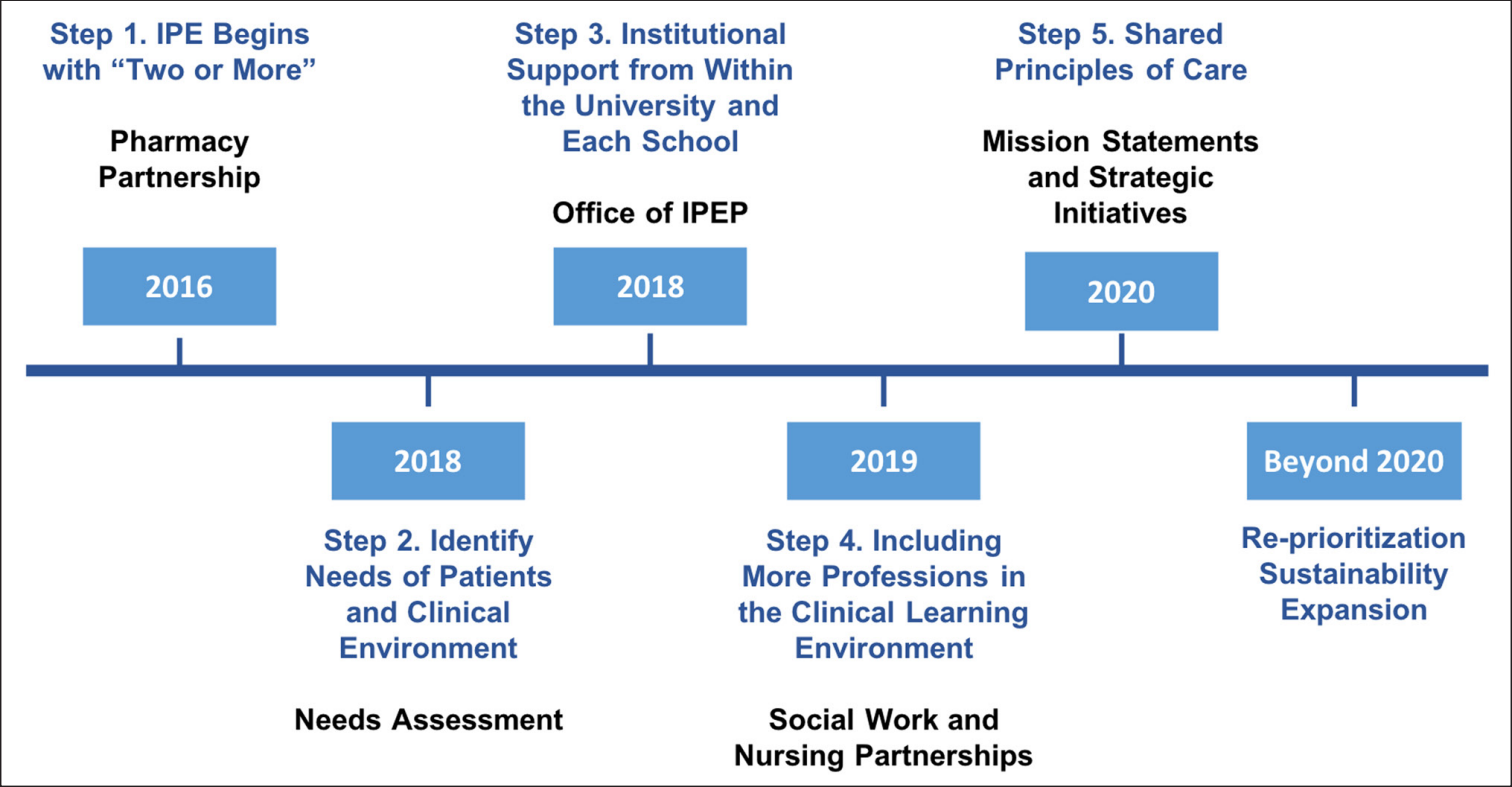
Steps to Collaboration.

The transformation of current educational approaches to quality interprofessional education and collaborative practice models requires patience, effort, time, and planning. As such, we lay out the steps which helped our team build sustaining interprofessional partnerships between oral health, pharmacy, and social work fields, and the initial collaborative efforts with nursing. These steps are also summarized in ***Figure 1***. Although this manuscript details the early process components for creating interprofessional and integrated services within clinical and didactic aspects of the ASOD, we also include lessons learned throughout this process and programming pivots made due to the COVID-19 pandemic. We will share directions to measure impact and outcome data in subsequent publications.

#### Step 1. Start Small, Work with Another Unit

In 2016, the UNC Eshelman School of Pharmacy and Adams School of Dentistry created a joint clinical pharmacist faculty position. The position was created to provide more in-depth consultation services in the student dentist and faculty practice environments as the first formalized collaborative partnership. A detailed description of the impetus for this partnership and the oral health considerations for medication management in care transitions has been previously published [39]. This new joint faculty position grew out of (a) observations made in dental student clinics that many patients had unaddressed medication needs and discrepancies, (b) a desire to increase interprofessional interactions for dental student learners, and (c) calls from dental faculty and students for opportunities to learn about the clinical applications of pharmacology and the impact of medications on oral health. Initial pharmacy interventions in the dental student clinics focused on diabetes care, but quickly adapted and expanded as the clinical pharmacist often noted other significant chronic conditions needing medication treatment that were not always accurately recorded in the health record, either due to omission, incomplete dosing, dated information, and/or duplications. With the support of both Schools, the joint clinical pharmacist faculty position was established after meetings between the Schools’ leadership, negotiation of appropriate full time equivalent (FTE) distribution, and the drafting of position responsibilities to fulfill aspects of scholarship, teaching, and clinical service in order to build and sustain the partnership.

As interprofessional pharmacy and dental services have continued to expand, so too did the number of consultation services personnel and pharmacy learners involved. These services have now grown to include two to three additional pharmacists for one half-day clinical allotment each and pharmacy doctoral students who provide a range of services: reviewing patient medication histories and profiles, providing medication and disease state education, documenting medications impacting oral health, counseling for prevention and treatment of medication-related oral manifestations, and answering medication-related inquiries. To help with this integration of pharmacy services into dental clinic settings and streamline services, a pharmacy consultation guidance document was created by the joint-appointed clinical pharmacist faculty to help dental providers and students determine when to consult a pharmacist. The joint-appointed clinical pharmacist faculty and student pharmacists are now fully involved in the didactic dental curriculum in teaching different aspects of pharmacotherapy and enhancing dental students’ learning about clinical applications of pharmacology as well. By starting with this partnership between two professions, we were able to identify (a) the respective roles and responsibilities that each profession would bring to an interprofessional care setting, (b) patients’ needs for integrated care, and (c) the opportunities the clinical environment provided to support expanding involvement of other health professions at the SOD. At the time of this publication, thirty-five student pharmacists have completed a clinical rotation of either four or eight weeks in this setting.

#### Step 2. Identify Needs of Patients and Clinical Learning Environment

In 2018, a nursing graduate student and a dental school faculty member conducted an internal needs assessment of the dental school in order to better understand the dental patient population’s overall health needs. While anecdotally it is known that dental clinics based in dental schools become safety nets for underserved populations, many of whom have multiple health, social, psychological, and pharmacological needs, there was a need to fully understand the specific population coming from North Carolina. This assessment reviewed local clinic data and select stakeholder interviews (i.e., with students, faculty, and staff) to identify service gaps and to determine why more comprehensive care was necessary for patients with high health and social needs. Health records of ASOD patients seen from 2012–2017 specifically in the dental student and dental hygiene student clinics – including self-reported health complaints taken from health history questionnaires – were cleaned and analyzed (***Table 1***).

**Table 1:** Summarized Patient Factors in Dental Student Clinics from Internal Needs Assessment*.


Demographics		

**Age in years (range)**	57 (14–100)	

**Sex**		

Male	46.5%	

Female	53.5%	

**Smoking Status (% Yes; average patients/year)**		

Current	13%	415 patients/year

Ex-smoker	35%	1065 patients/year

Never smoked	52%	1602 patients/year

**Health History Condition Questions (% Yes)**		

Physician care past 2 years?	78.2%	

High blood pressure	55.4%	

High cholesterol	36.6%	

Heart disease	10.0%	

Diabetes	15.8%	

Cancer or tumors	18.2%	

Inflammatory diseases	33.7%	

Frequent headaches or sinus problems	18.7%	

Asthma	11.7%	

Thyroid problems	14.2%	

Hepatitis or liver disease	3.7%	

Blood disorders	7.0%	

Kidney problems	7.7%	

Stomach/intestinal problems	24.8%	

Severe anxiety or depression	22.4%	

Radiation, surgery, chemotherapy	13.0%	

Heart attack, stroke, bypass	9.0%	


* Patient self-reported.

During the five-year period assessed, the ASOD accommodated almost 36,000 patient visits annually; the mean age of patients seen in the clinics was 57 years; and patients’ ages ranged from 14 years to over 90 years. Approximately 22% of these visits were made by patients who had neither seen nor had contact with a primary care provider in the past two years. Notably, the most common chronic conditions affecting the dental patient population were also the most common chronic conditions affecting North Carolinians broadly. Over 55% of visits were made by patients with high blood pressure; 36.6% of visits were made by patients reporting high cholesterol levels; and 33.7% of visits were made by patients reportedly suffering from inflammatory diseases. Nearly 25% of patient visits were with persons suffering from stomach or intestinal problems or high rates of severe anxiety or depression. Patterns of patients with uncontrolled conditions being screened out of receiving care were noted in data from interviews and observations. In the general dentistry clinics, elevated glucose and elevated blood pressure were often responsible for changes to the treatment plan that was intended to be administered during the scheduled appointment. Clearly, the internal needs assessment found ample evidence of service gaps and opportunities to address those gaps in integrated care settings.

At the same time, the University’s professional programs were taking efforts to identify clinical learning opportunities for students at new field placement or practicum sites. Although many area resources for clinical sites have already been utilized, key faculty from the involved schools recognized that expanding clinical learning opportunities on campus – including at the ASOD – would allow other professions (e.g., pharmacy, nursing, and social work) to participate in patient care and address patients’ other presenting needs beyond the scope of oral health.

#### Step 3. Institutional Support from Within the University and Each School

Nationally, 139 schools to date have implemented similar institution-wide interprofessional education and practice models [40]. Although the scope and reach of these programs vary, our institution did not have a formal procedure for establishing this cross-campus collaboration. Accordingly, in response to our efforts, the Office of Interprofessional Education and Practice (IPEP) was established in 2018 [41]. As noted in the Health Professions Accreditors Collaborative (HPAC) guidelines, institutional support from the highest levels of a University signal to health affairs and professional schools the importance of interprofessional collaboration in which student learners from different health disciplines train side by side in clinical settings [42]. Once the Office of IPEP was established, each dean from the participating schools and departments appointed a faculty member to represent their schools as an IPEP director. This new formal structure provided faculty with systematic channels for collaboration. Although our institution had a rich history of interprofessional education opportunities [43,44], the new IPEP directors shared a vision of building interprofessional clinical learning environments that comprehensively met clients’ care needs and provided interprofessional learning environments beyond didactic coursework.

#### Step 4. Including More Professions in the Clinical Learning Environment

Once the ASOD’s collaboration between the UNC Eshelman School of Pharmacy was successfully established, we used a scaffolding approach to incorporate the UNC School of Social Work and UNC School of Nursing into clinical and didactic programs at the ASOD. One of the biggest barriers to including more professions into the ASOD clinical faculty is that these positions did not yet exist, meaning that students often did not have a clear professional role model in this new setting. The lack of professional representation from students’ respective schools also meant that students lacked the required and appropriate person to supervise their clinical learning and required field hours as mandated by each professions’ accrediting body.

To rectify this issue, the deans of each participating school (dentistry, pharmacy, nursing, and social work) and the assistant provost of the Office of IPEP held a joint meeting to garner support for increasing interprofessional collaboration. Additionally, the ASOD expanded the number of participating faculty who specialized in oral medicine, the medical specialty of dentistry. These individuals have vast training and experience working in multidisciplinary, interprofessional teams, therefore their input and guidance led to a natural improvement of ASOD’s ability to plan how to successfully provide integrated services with the other disciplines. Faculty and leadership support led to the creation of a joint faculty appointment between the ASOD and the School of Social Work: a social work coordinator position to be filled by a clinical social worker.

The basis for this inaugural position was crafted from previous research a team member had done on the multi-faceted roles of social workers in integrated primary care settings which included behavioral health expertise, care coordination, and linkages to community resources and support [45]. Further, extant literature illuminated how social workers have been integrated into dentistry services to improve patient care yet these models pre-dated the shift to integrated models of care [47,48]. More recent literature described the role social workers play on interprofessional teams to address dental caries [46]. Therefore, with these examples in mind, the inaugural social work care coordinator’s responsibilities included: (a) providing behavioral health and care management interventions to patients in need; (b) supervising MSW student interns as field education is a considerable component of social work education; (c) teaching one course in the School of Social Work and guest lecturing in the ASOD on topics including patient communication, the social determinants of health and healthcare access and policies; and (d) engaging with collaborators across campus and the community. In the first six months of the clinical social work faculty’s term, two MSW students were selected for field placements in the ASOD, where they would each complete over 500 hours of field internship work.

We took a different approach to incorporating School of Nursing staff and students into the ASOD’s interprofessional curriculum and services. Data from a newly integrated electronic health record and findings from the ASOD’s internal needs assessment indicated a need for more dental services incorporating primary care. For this reason, the ASOD and School of Nursing created a joint faculty appointment focused on incorporating the services of an occupational health nurse practitioner who could serve both patients’ acute health needs and dental school staff’s needs. This position’s responsibilities included: (a) evaluating patients for acute, chronic, and/or wellness visits (including faculty, staff, students, community, or dental patients with acute illnesses that benefitted from treatment on the day of the visit); (b) performing health screenings and point-of-care testing; (c) delivering vaccines; (d) helping establish care for patients without any primary care in the UNC Health System or in their community of origin; and (e) providing consultations and referrals for medically complex patients.

#### Step 5. Shared Principles of Care

In drawing together these different health disciplines, establishing a shared purpose was crucial to determining the best way to establish initial patient care projects and promote joint student learner engagement. Once all new joint faculty were hired, we developed a vision and mission for the joint collaborations as well as logic models for prioritizing target outcomes overall and within each discipline. We jointly created a mission statement with the overall goals of improving access to primary care and oral health services, initiating referrals to primary care, providing dental services to achieve whole health, and providing continuity of care for the patients served. This mission statement was informed by two core tenets: there is no wrong door to entry into the healthcare system and each clinical environment should be equipped to best serve patients’ whole health needs. We clarified the roles and responsibilities of all involved parties, as shown in ***Table 2***. For initial implementation, integrated care efforts focused on the ASOD’s Admissions Clinic (one of several dental student practice clinics), which serves as the entry point for patients coming to receive care at the ASOD. With support from the Office of IPEP, team members jointly created strategic initiatives for community, education, and impact. Areas for target outcomes of the collaboration included comprehensive care, coordinated and accessible care, patient-centered care, student engagement, and continuous quality improvement and safety.

**Table 2:** Health Discipline Roles for Integrated Health Services.

Health Discipline	Roles

**Clinical Social Worker**	Provides direct patient care through psychosocial assessment, intervention, and care coordination in order to improve patient treatment outcomes. Collaborates with community partners in order to improve the patient referral experience and enhance the relationships patients have within their home communities. Consults with dental and oral hygiene students on gathering psychosocial health information and using effective communication skills (i.e., managing patients’ anxiety or fears, motivational interviewing skills, among others). Serves as advocates for patients’ needs and considers how social factors are influencing treatment and care plans.

**Nurse Practitioner**	Provides student and patient consultations on acute and chronic disease management, patient education, and assistance with primary and specialty referrals. Promotes convenient and approachable access, which enhances the school’s ability to meet both non-urgent oral and physical health needs.

**Oral Medicine**	Provides oral health care of medically complex patients and diagnoses and treats the oral ramifications of systemic disease. Provides medical management of neurologic conditions, dermatologic conditions and pain syndromes that affect the orofacial region. Provides care before, during, and after cancer treatment to reduce or prevent the incidence of complications related to cancer or its treatment.

**Pharmacist**	Provides student, faculty, and patient consultations on comprehensive medication management, impact of medications on oral health, acute and chronic disease management education, patient education, and assistance with referrals for primary care and medication access. Consults with dental and hygiene students to create complete and accurate home medication lists and recommend safe medication use practices.


## Discussion

Despite prior efforts to establish quality interprofessional education opportunities [42], there is no guidebook on how to effectively establish them. Individual clinician’s (sometimes mistaken) beliefs that they are already practicing collaboratively, hierarchal differences in professional power, knowledge gaps that impact care delivery, and professional culture are all identified barriers to the implementation of interprofessional practice [49]. Although we fortunately did not encounter these barriers while establishing collaborative partnerships between the Schools of Dentistry, Pharmacy, Social Work, and Nursing, the implementation and sustainability of these partnerships were challenged by the rollout of a new electronic health record (EHR) system, the COVID-19 pandemic, and related clinical and financial issues caused by both of those changes.

In February 2020, integration of a new EHR that had historically only been used in larger health systems led to school-wide changes in clinical workflow, access to patient information, and student engagement. This system change brought benefits, including increased access to patient health information and the ability to collect more information to inform clinical decisions. In the ASOD, the implementation of the EHR has improved communication between primary, specialty care providers, and patients – especially patients already seeing providers who utilized the same EHR system – and opened new opportunities to expand the continuity of care as well as gain more comprehensive information on patients’ health. However, implementing the new EHR also introduced some challenges, such as the need to identify gaps in technology support, better adapt workflows to new technology, and ensure consistency among the EHR’s users (i.e., students, faculty, and clinical staff) to increase its efficiency and utilization. The newness of this important health system, which has immense promise, has not yet been fully realized. As utilization of the EHR becomes more efficient, we expect that collaboration across disciplines through the EHR will increase.

In March 2020, the impact of the COVID-19 pandemic rippled across the University and led to the closure of all student practice clinics for five months. This closure greatly impacted our initial focus on integrated care services in the SOD Admissions Clinic as well as the momentum of all the joint clinical faulty working together to provide coordinated services. During this time, the clinical faculty and health discipline leaders met regularly to further plan services by developing and refining a logic model for integrated service delivery, identifying roles and responsibilities (***Table 2***), and devising strategies for adapting to the changing clinical environment. One success of these meetings was the implementation of a pilot telehealth model that included social work and pharmacy faculty and students from pharmacy, social work, and dentistry. In this model, students conducted pre-visit video-conference calls with patients, who then had tele-dentistry visits to address dental care needs. Determining how best to engage dental student learners in this model has been an ongoing process.

The COVID-19 pandemic has also had unforeseen financial implications that have altered the make-up of the schools’ collaborative partnerships. Specifically, this included a significant decision to re-allocate the nurse practitioner faculty position elsewhere, changing the originally planned (i.e., pre-COVID) composition of the interprofessional team and producing workflow changes and turnover in leadership and staff. As a result, the initially planned strategic initiative of coordinated care with nursing services has been temporarily discontinued. As with any team, when changes to team structure occur, there must be a re-evaluation of all team members’ roles and responsibilities [50].

A key lesson of this disrupted rollout has been that partnerships exist in dynamic environments, requiring partners to plan accordingly. The clinical faculty and leadership of participating schools are continuing to adapt to the changing healthcare environment in order to improve patient care and student learner preparation for future clinical environments. Although still incipient, the integrated clinical services are still expanding and the engagement of students beyond the ASOD has remained high throughout 2020–2021, including eleven doctor of pharmacy students, two master’s in social work interns (the first ever in this setting), and two public health nursing students. SOD dental and dental hygiene students and clinic directors also remain engaged as we implement curricular transformations in response to COVID-19 and accreditation needs [44].

## Conclusion

Integrated care in the dental clinic environment is just as important as integrated care in any clinical environment. This paper details the steps taken, strategies developed, and lessons learned in one academic institution over the process of promoting integrated care and interprofessional education in clinical learning environments housed in a school of dentistry.

In coming years, we will expand the number of student learners in this setting from across professional schools within our university. As quality clinical learning environments can be difficult to secure and sustain, this model allows for our university to create increased clinical opportunities for students within our own institution. Further, we will continue to measure and improve patient care, educational opportunities, and organizational outcomes of these collaborative partnerships. Efforts to evaluate this integrated care model in terms of quality improvement process measures and patient outcomes and to assess student’s growth and development of required competencies across professional disciplines will soon begin. Patient satisfaction and experiences with the interprofessional team is another metric that will be used in future evaluation efforts. Further, with EHR data now being collected more systematically, screening and assessment tools can capture pre and post intervention changes, count occurrences of specific interventions, and report documentation practices among the interprofessional care team. Although this interprofessional model included multiple types of providers, future growth could also increase collaboration with other disciplines (e.g., nutrition or speech language pathology). Given the recognized benefits of integration, oral health must be understood and utilized as an essential component of integrated services. This paper highlights the potential that exists when academic units partner together to provide clinical care and train future providers. As the health system continues to change, efforts to integrate physical, behavioral, and oral health services show great promise and potential for these fields’ future workforces and clients.
